# A description of the relationship in healthy longevity and aging-related disease: from gene to protein

**DOI:** 10.1186/s12979-021-00241-0

**Published:** 2021-06-25

**Authors:** Xiaolin Ni, Zhaoping Wang, Danni Gao, Huiping Yuan, Liang Sun, Xiaoquan Zhu, Qi Zhou, Ze Yang

**Affiliations:** 1 The Key Laboratory of Geriatrics, Beijing Institute of Geriatrics, Beijing Hospital, National Center of Gerontology, National Health Commission; Institute of Geriatric Medicine, Chinese Academy of Medical Sciences, Beijing, 100730 P.R. China; 2 Graduate School of Chinese Academy of Medical Science and Peking Union Medical College, Beijing, 100001 P.R. China; 3 Peking University Fifth School of Clinical Medicine, Beijing Hospital, Beijing, P.R. China

**Keywords:** Longevity, Genetic characteristics, Homeostasis, Cardiovascular disease, Alzheimer’s disease

## Abstract

Human longevity is a complex phenotype influenced by both genetic and environmental factors. It is also known to be associated with various types of age-related diseases, such as Alzheimer’s disease (AD) and cardiovascular disease (CVD). The central dogma of molecular biology demonstrates the conversion of DNA to RNA to the encoded protein. These proteins interact to form complex cell signaling pathways, which perform various biological functions. With prolonged exposure to the environment, the in vivo homeostasis adapts to the changes, and finally, humans adopt the phenotype of longevity or aging-related diseases. In this review, we focus on two different states: longevity and aging-related diseases, including CVD and AD, to discuss the relationship between genetic characteristics, including gene variation, the level of gene expression, regulation of gene expression, the level of protein expression, both genetic and environmental influences and homeostasis based on these phenotypes shown in organisms.

## Backgroud

**Fig. 1:**
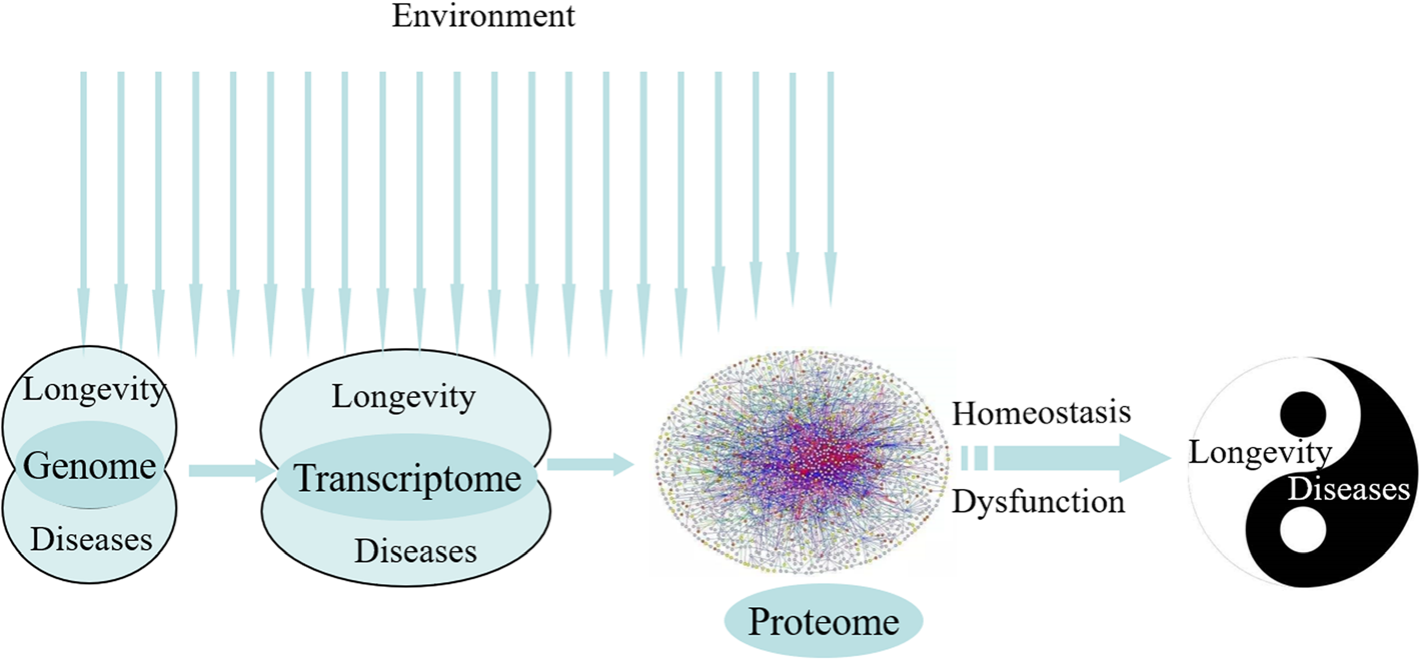
The process of longevity and aging-related diseases formation

Since time immemorial, humans have desired longevity, as evidenced by inscriptions found in ancient Egypt and even in older Sumerian tablets [1]. Both genetic and environmental factors are known to affect the human life span. Studies have shown that people with a longer lifespan generally delay or postponement occurrence of these major diseases that develop with age, such as metabolic diseases (diabetes, cardiovascular disease (CVD), and neurodegenerative disorders), even escape them [2]. Studies have shown that longevity and resistance to diseases are mediated by common mechanisms, and the genetic factors related to longevity and aging-related diseases have been speculated to have an inverse relationship [3, 4], but need to discuss from more levels (Fig. 1).

CVD affects the heart and its blood vessels, causing myocardial infarction, heart failure, and stroke; it constitutes one of the primary causes of death worldwide [5]. Metabolic dysfunction in the microenvironment of organisms is a fundamental core mechanism underlying CVD. The metabolic disorders which are caused by genetic or environmental factors are the main internal risks that induce CVD with advancing age. For example, *SIRT5* mediates lysine desuccinylation of proteins those participated in short-chain fatty acid-associated metabolic pathways. Mice that knock out the *SIRT5* gene show reduced cardiac function and develop hypertrophic cardiomyopathy with aging [6]. Obesity which energy intake exceeding energy expenditure, and then induced a series of metabolic derangements is one of the important risk factors for cardiovascular disease (CVD) [7].

Alzheimer’s disease (AD) constitutes a chronic neurodegenerative condition that eventually leads to dementia. Individuals with AD experience noticeable symptoms, such as memory loss and language problems that affect their quality of life. Its fatality rate and pathogenic mechanism differentiate it from CVD. With the increasing population aged > 65 years, there has been a dramatic increase in the number of patients with AD making it the sixth leading cause of death in the USA [8]. The pathogenic mechanism of AD involves the formation of intracellular neurofibrillary tangles (NFTs) and extracellular Aβ or senile plaques (SPs) in the brain. Studies have found a link between Apolipoprotein E (APOE) and the onset of AD. Furthermore, altered cholesterol levels have also been shown to induce AD by increasing Aβ formation [9].

Traditionally, longevity and aging-related diseases are considered contradictory states. Based on their pathogenic mechanisms, aging-related diseases can occur either due to (1) abnormal cellular microenvironment, which induces diseases indirectly (CVD), or (2) abnormal gene coding proteins, which induce diseases directly (AD). However, regardless of longevity, CVD or AD are known to result from genetic and environmental interactions. Therefore, in this review, we focused on these three representative phenotypes, longevity, CVD, and AD, to analyze the process of phenotype formation. We aimed to illustrate the internal relationship between longevity and aging-related diseases for future applications.

## Gene variation

Based on the conventional genotype-to-phenotype theory, the phenotype trait is determined based on the synchronized interaction between encoded protein shape and genetic expression. This implies that genes regulate phenotypic expression; however, there is no direct relationship between genes and traits [10].

Longevity-related genome-wide association studies (GWAS) have verified the role of approximately 57 gene loci. A 1994 study on centenarians first implied the role of the *APOE* gene in longevity, with a low and high frequency of the Ɛ4 and Ɛ2 alleles, respectively [11]. The frequency of *ApoE* Ɛ4 and Ɛ2 alleles were also replicated among centenarians compared with their ethnically-matched younger controls in different cohorts [12]. Subsequently, several GWAS analyses discovered various genetic loci associated with longevity, such as *TOMM40*, *APOC1*, *SOD2*, *KL*, *CDKN2B*, *ANKRD20A9P*, etc. [2, 13, 14]. Our previous genetic studies on longevity in China have shown that *FOXO3*, *CETP*, *SIRT1* and *HLA-DQ* genes were associated with the phenotype of longevity [15–18].

**Fig. 2:**
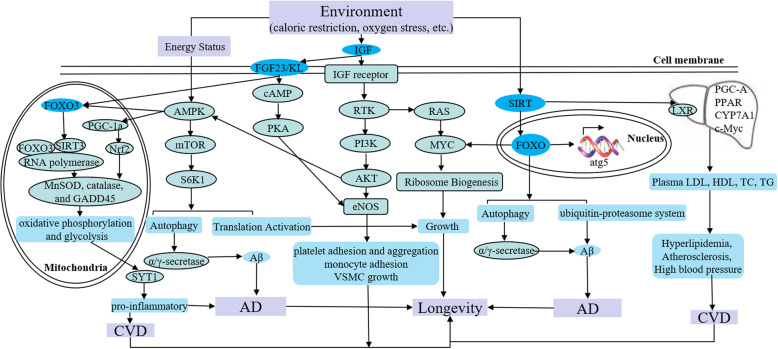
The molecular relationship of longevity and aging-related diseases. Environmental factors activate insulin/IGF-1/PI3K, AMPK/mTOR, SIRT/FOXO, and IGF/RTK/MYC multiple pathways. At the same time, these pathways interact with each other to regulate the molecular signaling cascade involved cytoplasm, mitochondria, and nucleus. Finally, the phenotype of longevity or aging-related disease is formed by the difference of molecular expression regulation in the same pathway. IGF: insulin like growth factor; RTK: receptor tyrosine kinase; PI3K: phosphatidylinositol 3-kinase; FOXO: forkhead box O; AMPK: AMP-activated protein kinase; mTOR: mechanistic target of rapamycin; S6K1: S6 kinase 1; MnSOD: manganese superoxide dismutase; PGC-1: peroxisome proliferator-activated receptor γ coactivator 1; Nrf2: NF-E2-related factor 2; SIRT, silent mating type information regulation 2; LXR: liver X receptor; atg5: autophagy-related 5; Aβ: amyloid-beta; eNOS: endothelial NO synthase; KL: Klotho; VSMC: vascular smooth muscle cells; SYT1: synaptotagmin 1

*FOXO3* encodes a transcription factor that regulates stress response, metabolism, resistance, cell cycle arrest, and apoptosis, and thus significantly impacts age-related phenotypes. The rs2802292 of *FOXO3* has been found to be significantly related to human longevity [19]. Additionally, longevity-related single nucleotide polymorphism (SNP) rs2802292*G allele of *FOXO3* has been shown to act as a protective factor against mortality due to CVD based on multivariate Cox regression models. A study used population-attributable risk (PAR) models to study Japanese, White, and Black Americans and found that the nonprotective TT genotype added 15, 9, and 3%, respectively, to the fatality risk of coronary artery disease (CAD) [20]. Recent studies showed that rs2802292*G allele is recognized and bound by HSF1 which is an evolutionarily highly conserved transcription factor, while the T-allele fails to do so. HSF1 induces the interaction of a promoter-enhancer interaction by chromatin looping at 5′ UTR of *FOXO3* and the rs2802292 region to increase *FOXO3* expression. and then activate the insulin/IGF-1/PI3K signaling cascade to affect antioxidant, metabolic, and DNA repair transcriptional programs, leading to increased tolerance to nutrient, genotoxic, and oxidative stress [19] (Fig. 2).

Klotho (*KL*) is a transmembrane protein that protects against cognitive decline as well as other aging-associated phenotypes. *KL-*VS, a functional haplotype, results from the perfect linkage disequilibrium between two *KL* missense variants (F352V [rs9536314] and C370S [rs9527025]). Compared with individuals who are homozygotes for the major/minor alleles (*KL-VS*^HET−^), individuals who are heterozygous for *KL-*VS (*KL-VS*^HET+^ status) exhibit increased serum levels of KL, resulting in healthy aging and longevity. Another study on 20,928 participants showed that individuals > 60 years who had *APOE* Ɛ 4 mutation with *KL-VS*^HET+^ genotype had a reduced risk for AD (*p* = 7.4 × 10^− 7^, OR = 0.75, 95% CI: 0.67–0.84), and this was more prominent between 60 and 80 years of age (*p* = 3.6 × 10^− 8^, OR = 0.69, 95% CI: 0.61–0.79). A significantly protective interaction was observed between *APOE* Ɛ 4 status and *KL-VS*^HET+^ status for AD risk in the group aged 60 to 80 years (*P* = 3.9 × 10^− 4^, OR = 0.76, 95% CI: 0.66–0.89) [14]. Numerous studies in the last 20 years have demonstrated that AD is mainly caused by the neurovascular toxicity of aberrant Aβ deposition in the brain. Studies in mice have shown that the upregulation of Klotho expression could inhibit insulin-like growth factor-1/AKT/mTOR signaling and then improve autophagy-induced Aβ_1–42_ clearance in vivo and in vitro to prolong lifespan [21] (Fig. 2).

## The level of gene expression

A useful approach for the identification of molecular markers of aging involves the analysis of changes in gene expression in the elderly. In humans, chronological age-based changes in gene expression have been identified in the brain, skeletal muscle, and dermal fibroblasts that reflect physiological aging via whole-genome expression profiling [22]. Thus, quantitative assessment of gene expression can explain the drastic phenotypic effects, such as changes in morphology and lifespan, resulting from changes in gene expression [23, 24].

The DAF-2 insulin/IGF-I receptor mediates the insulin/IGF-I signaling. A study showed that *C. elegans* daf-2 mutants with reduced DAF-2 activity lived approximately twice as long as the wild-type animals [25]. Also, Cohen et al. showed that knockdown of *DAF-2* in *C. elegans* reduced Aβ_1–42_ toxicity to reduce AD risk [26].

Insulin signaling is known to stimulate glucose uptake and regulate glucose homeostasis. Studies have shown that increased lifespan has been observed in organisms with weakened insulin/IGF-1 signaling due to genetic alterations [27]. A cross-sectional study found elevated levels of serum IGF-1 during the early stages of severe AD. And then a study in aging brains indicated that chronic neuronal IGF-1 stimulation increased β-secretase activity leading to Aβ accumulation by a change of expression from TrkA to p75^NTR^ [26]. Additionally, studies have linked low levels of serum IGF1 to vascular changes in aging and obesity as well as to increased cardiovascular morbidity and mortality. Animal studies in vivo and in vitro have shown that IGF1 induces vasodilatation by producing nitric oxide (NO), reduces endothelial dysfunction, promotes mRNA expression of specific contractile proteins, improves myocardial contractility, and limits ischemia-reperfusion injury to maintain normal cardiovascular health [28, 29] (Fig. 2).

## Regulation of gene expression

The central dogma of molecular biology describes the conversion of DNA to RNA to protein [30]. Studies have shown that protein-coding genes account for approximately 1.5–3% of the whole human genome [31]. Only a few genes are activated, and their transcription, translation, and post-translational modifications result in various proteins and peptides [32]. Therefore, regulation of gene expression via DNA methylation, histone modifications, promoter and enhancer interactions, pre-mRNA splicing, and noncoding RNAs facilitate the differential utilization of genetic information, providing a defined phenotype and function [33].

Generally speaking, gene-expression patterns is achieved by enabling or restricting the transcriptional potential of genomic domains through DNA methylation, histone modification and chromatin regulation [34]. The DNA methylation data is used as aging biomarkers (“epigenetic clocks”) to accurately estimate age. “Horvath epigenetic clocks” as a classical biological age prediction clock, selected 353 CpG sites of which the methylation status is associated with age. These genes are involved in some main cell functions, such as cell death and survival, cell growth and proliferation, organismal and tissue development, and cancer [35]. Yet histone modifications are key epigenetic regulators that affect gene expression, cellular phenotypes, and lifespan by regulating chromatin structure and gene transcription [36]. One study suggested that increased lifespan was achieved by disrupting H3 acetylation on K9 and K18 residues, which depended on Gcn5 as well as the linked protein Ngg1 [37]. Histone deacetylases (HDACs) are histone-modifying enzymes in the cardiovascular system. Studies have shown that inhibition of HDACs can protect against CVDs by preventing vascular smooth muscle cell growth (with implications for atherosclerosis), reducing hypertension, ameliorating ischemic/reperfusion injury and postischemic remodeling, and blocking cardiac hypertrophy in the case of heart failure to prolong lifespan [38]. Recent studies have attempted to analyze the effect of gene regulation on 3D chromatin domains and found a correlation between evolutionarily conserved Topologically Associating Domains (TADs) and gene expression by regulating the interaction between promoters and enhancers [39]. Studies have shown the presence of various regulatory elements in the non-coding sequences of the genome, including changes of spatial structure in Lamina Associated Domains (LADs), which could result in age-related diseases [40], resulting in reduced life expectancy.

Pre-mRNA splicing involves the removal of introns and joining of exons to form a mature mRNA. Recent studies have confirmed that pre-mRNA splicing is involved in pro-longevity processes. A study on patients with AD identified that 1174 exons underwent splicing with age in the human temporal cortex, of which 95% changes occurred in frontotemporal lobar degeneration (FTLD) [41]. On other side, noncoding RNAs (ncRNAs) produced after pre-mRNA splicing. Although ncRNAs do not encode proteins and are divided into small ncRNAs (sncRNAs, 18 ~ 200 nt) and long noncoding RNAs (lncRNAs, > 200 nt), ncRNAs regulate almost all cellular functions, such as proliferation, apoptosis, autophagy, and cell cycle control by regulating gene expression [42]. Additionally, age-regulated lncRNAs can fine-tune the regulation of longevity-related proteins by quantitatively regulating mTOR expression. The overexpression of miR-34c, which belongs to sncRNAs in the hippocampus of young mice and is regulated by ROS-JNK-P53 pathway, has been shown to result in age-related memory impairment. Experiments in vitro showed that the inhibitor of miR-34c rescued the Aβ-induced decrease in primary hippocampal neurons by post-transcriptional regulation of SYT1 expression during AD development [43, 44] (Fig. 2).

## The level of protein expression

Proteostasis involves the maintenance of a functional equilibrium between protein synthesis, fidelity, folding, localization, modification, and degradation and plays an important role in health and longevity. The progressive loss of proteostasis is a hallmark of aging since age-related diseases and conditions are associated with the inability of the cell to maintain healthy proteins or eliminate defective proteins, as observed in neurodegenerative diseases, cardiac dysfunction, cataracts, and sarcopenia [45].

Myc is a highly conserved helix-loop-helix leucine zipper transcription factor that promotes metabolism, cellular growth and proliferation, as well as enhanced energy production. Also, Myc positively regulates ribosome biogenesis to increase protein synthesis, while reducing translation can extend lifespan. Compared with Myc +/+ mice, Myc +/− mice have been found to have a reduced fatality rate in both sexes and across all age groups by modulating IGF-1/AKT/mTOR signaling cascade (Fig. 2). Additionally, FOXO1 as a critical checkpoint of endothelial growth, promotes endothelial quiescence by antagonizing MYC, which leads to a coordinated reduction in the proliferative and metabolic activity of endothelial cells (ECs). The reduction in oxidative metabolism induced by FOXO1 to minimize the production of mitochondria-derived ROS, thereby conferring protection against the high-oxygen environment to prevent age-associated diseases and promote longevity [46–50].

## Importance of genetic and environmental factors

Longevity depends on complex interactions between various genetic and environmental factors. In particularly, epigenetics is adjusted and changed with the stimulus of the environment, so it is difficult to put aside the influence of environment on lifespan. The environment is divided into internal and external systems.

Microbiomics, the internal environment system, aims to identify and analyze the microbial genome constituents, characterize host-microbiome interactions, and determine its influence on health and diseases [51]. This dynamic balance is maintained by suppressing immune responses to commensal bacteria (the P-glycoprotein [P-gp]/endocannabinoid axis) and activating inflammatory responses to pathogens or aberrant signaling molecules (multidrug-resistant protein 2 [MRP2]/hepoxilin A3) to ensure a longer lifespan; this balance can be disturbed by a dysbiotic microbiome. Compared with patients without dementia, patients with AD were found to induce lower P-gp expression levels, possess fewer butyrate-synthesizing bacteria and higher abundances of taxa that are known to cause proinflammatory states. Therefore, the intestinal microbiome modulated intestinal homeostasis by increasing in inflammatory, and decreasing in anti-inflammatory, microbial metabolism to reduce the occurrence of AD [52]. A study analyzed the effect of microbiome-diet-inflammation pattern on the probability of development of CVD and identified 48 microbial pathways associated with CVD risk that affected lifespan by interleukin members (IL-10, IL-6, IL-12P70 and IL-18 bp) involved in bacterial AA biosynthesis, GDP-mannose metabolism glycolysis and homolactic fermentation. Therefore, there may be a possible interaction between the host and the gut microbiome through glycose metabolism [53].

The external environment system can also produce physiological and developmental changes resulting in alternative phenotypes. For example, there exists a direct correlation between increased lifespan and better living conditions [54]. Better living condition includes healthy nutrition, less oxidative stress, healthy life style, taking exercises and many other environmental factors, etc. Diet restriction affects various factors, such as SIRT1, AMPK, and mTOR, that influence cellular processes, apoptosis, etc., and promote aging [13]. Reduced food intake, avoiding malnutrition has been shown to ameliorate aging-associated diseases and extend human lifespan by IGF-1/mTOR network [55] (Fig. 2). A low-calorie diet might slow or prevent the pathogenesis and progression of AD through SIRT1-mediated deacetylation of FoxO to inhibit Rho-associated protein kinase-1 (ROCK1) and consequently increase α-secretase activity and decrease Aβ production in autophagy pathways [56]. Healthy diet interventions promote weight loss, preventing metabolic syndrome, and reducing the development of CVD [57]. Oxidative stress is also a major characteristic of longevity or aging-related diseases. Almost all of the diseases such as CVD, hypertension, cancer, and AD that limit longevity and healthful aging are associated with increased oxidative stress [58]. The production of oxidants from multiple sources is increased, and the adaptive response of organisms to oxidative stress and the ability of repair systems will decline with aging by Nrf2/EpRE signaling system [59] (Fig. 2).

## Protein networks regulate homeostasis to influence lifespan

No matter mutated genes, abnormal regulation of gene expression, or environmental factors, which affected the quality and quantity of protein productions, all impacted homeostasis of cells. “Homeostasis” does not imply rigidity of the dynamic system. Usually, certain internal variables remain approximately constant over a finite range of inputs corresponding to external variables through various regulatory mechanisms. Outside that range, the mechanisms cease to function, threatening the physical health [60].

In the face of calorie restriction and oxidative stress, glucose metabolism provided both substrates and metabolic control for insulin to promote fatty acid and glyceride–glycerol synthesis [61]. Lipid metabolism play an important role in various physiological and pathological processes. In humans, excessive fat storage in the form of triglycerides has been associated with diseases, such as CVD. Also, peripheral serum levels of lipids have been associated with AD and cognitive impairment by an allele of *APOE*. However, emerging studies in mammals have suggested that specific alterations in fat profiles and even elevated fat storage can be associated with longevity [62, 63].

Sirtuin 1 affects cholesterol and lipid metabolism via various proteins and transcription factors, including farnesoid X receptor (FXR), PGC-1α, cholesterol 7α-hydroxylase (CYP7A1), PPARα, and PPARγ, sterol regulatory element-binding proteins (SREBPs), and liver X receptor (LXR). A study found significantly decreased total plasma and LDL cholesterol levels in transgenic mice overexpressing *SIRT1*, while reduced HDL cholesterol levels were found in *SIRT1* knockout (Sirt1−/−) mice after an 8-day LXR agonist administration. This result suggested that overexpression of *SIRT1* decreased blood cholesterol levels and protected against CVDs [64] (Fig. 2). A deficiency in sirtuin 1 levels has been recently implicated in increased tau acetylation, a dominant post-translational modification and key pathological event in AD [65]. Also, sirtuin 1 was found to extend the lifespan by regulating numerous vital signaling pathways, including DNA repair and apoptosis, neurogenesis, mitochondrial biogenesis, glucose and insulin homeostasis, etc. [66].

Numerous studies have shown that *APOE* Ɛ4 is associated with a substantially decreased odds for extreme longevity and increased risk for death, while *APOE* Ɛ2 is associated with significantly increased odds for longevity, with decreased risk for death [67]. Unlike individuals with *APOE* Ɛ2, individuals with *APOE* Ɛ4 have lower plasma concentration of APOE and C-reactive protein (CRP), along with higher plasma levels of cholesterol, LDL-C, APOB, lipoprotein (a), atherosclerosis, and BMI, and are at a higher risk of CVD. Additionally, microhemorrhages in cerebral blood vessels might damage the associated neural tissue, resulting in the formation of amyloid plaques and tau protein tangles, leading to the onset of AD. It has also been shown that age-dependent changes in the levels of lipid and cholesterol in the brain play a critical role in longevity [68]. Thus, in multicellular organisms, the control of gene expression or gene variation is critical not only for development but also for adult cellular homeostasis [69].

## Conclusions

Recent studies have enhanced our understanding of the determinants of human lifespan and longevity. Additionally, various factors that influence longevity and aging-related diseases have been identified, along with the detection of the physiological sources of damage that cause aging-related diseases, the compensatory responses that try to repair homeostasis, and the possibilities of exogenous intervention to prevent aging-related diseases and promote healthy aging and longevity [70]. However, the intricate interplay between these components is still unclear.

In this review, we focused on two different states: longevity and aging-related diseases (CVD or AD), to discuss the relationship between genetic characteristics, including gene variation, the level of gene expression, gene expression regulation, the level of protein expression, both genetic and environmental influences, and homeostasis based on the formation of these phenotype in organisms. Environmental factors activate insulin/IGF-1/PI3K, AMPK/mTOR, SIRT/FOXO, and IGF/RTK/MYC multiple pathways. At the same time, these pathways interact with each other to regulate the molecular signaling cascade involved cytoplasm, mitochondria, and nucleus. Finally, the phenotype of longevity or aging-related disease is formed by the difference of molecular expression regulation in the same pathway. Longevity and CVD or AD, which are interconnected and opposites, are intrinsically related and mutually conditioned by the metabolism balance (Fig. 2). Our analysis indicated that the phenotypic variation in longevity or aging-related diseases in humans occurs due to the differences in genomes or gene expression regulation and the long-term effects of different environments; the in vivo homeostasis of metabolism is adjusted accordingly for adaptation (Fig. 1).

## Data Availability

This is a review which don’t involve databases and materials.

## Abbreviations

AD: Alzheimer’s disease
CVD: Cardiovascular disease
NFTs: Neurofibrillary tangles
SPs: Senile plaques
APOE: Apolipoprotein E
GWAS: Genome-wide association studies
SNPs: Single nucleotide polymorphisms
PAR: Population-attributable risk
KL: Klotho
HDACs: Histone deacetylases
TADs: Topologically Associating Domains
LADs: Lamina Associated Domains
FTLD: Frontotemporal lobar degeneration
ncRNAs: Noncoding RNAs
Sirt1: Sirtuin 1
FXR: Farnesoid X receptor
CYP7A1: Cholesterol 7α-hydroxylase
SREBPs: Sterol regulatory element-binding proteins
LXR: liver X receptor
CRP: C-reactive protein
HDL: High density lipoprotein
LDL: Low density lipoprotein
MnSOD: Manganese superoxide dismutase
IGF: Insulin like growth factor
RTK: Receptor tyrosine kinase
PI3K: Phosphatidylinositol 3-kinase
FOXO: Forkhead box O
AMPK: AMP-activated protein kinase
mTOR: Mechanistic target of rapamycin
S6K1: S6 kinase 1
PGC-1: Peroxisome proliferator-activated receptor γ coactivator 1
Nrf2: NF-E2-related factor 2
SIRT: Silent mating type information regulation 2
atg5: Autophagy-related 5
Aβ: Amyloid-beta
VSMC: Vascular smooth muscle cells
SYT1: Synaptotagmin 1
ROCK1: Rho-associated protein kinase-1

## References

[1] Labat-Robert J, Robert L (2015). Longevity and aging. Mechanisms and perspectives. Pathol Biol (Paris).

[2] Zeng Y, Nie C, Min J, Liu X, Li M, Chen H (2016). Novel loci and pathways significantly associated with longevity. Sci Rep.

[3] Franceschi C, Garagnani P, Olivieri F, Salvioli S, Giuliani C (2020). The contextualized genetics of human longevity: JACC focus seminar. J Am Coll Cardiol.

[4] Gunn-Moore D, Kaidanovich-Beilin O, Gallego Iradi MC, Gunn-Moore F, Lovestone S (2018). Alzheimer's disease in humans and other animals: a consequence of postreproductive life span and longevity rather than aging. Alzheimers Dement.

[5] de Jong L, Bobeldijk-Pastorova I, Erdmann J, Bijker-Schreurs M, Schunkert H, Kuivenhoven JA, van Gool AJ (2020). Sharing lessons learnt across European cardiovascular research consortia. Drug Discov Today.

[6] Chen XF, Chen X, Tang X (2020). Short-chain fatty acid, acylation and cardiovascular diseases. Clin Sci (Lond).

[7] Elagizi A, Kachur S, Lavie CJ, Carbone S, Pandey A, Ortega FB, Milani RV (2018). An overview and update on obesity and the obesity paradox in cardiovascular diseases. Prog Cardiovasc Dis.

[8] 2020 Alzheimer's disease facts and figures. Alzheimers Dement. 2020. 10.1002/alz.12068. Epub ahead of print. 10.1002/alz.12068 32157811

[9] Li X, Zhu X, Zhang W, Yang F, Hui J, Tan J, Xie H, Peng D, Ma L, Cui L, Zhang S, Lv Z, Sun L, Yuan H, Zhou Q, Wang L, Qi S, Wang Z, Hu C, Yang Z (2018). The etiological effect of a new low-frequency ESR1 variant on mild cognitive impairment and Alzheimer's disease: a population-based study. Aging (Albany NY).

[10] Nussinov R, Tsai CJ, Jang H (2019). Protein ensembles link genotype to phenotype. PLoS Comput Biol.

[11] Schächter F, Faure-Delanef L, Guénot F, Rouger H, Froguel P, Lesueur-Ginot L, Cohen D (1994). Genetic associations with human longevity at the APOE and ACE loci. Nat Genet.

[12] Santos-Lozano A, Santamarina A, Pareja-Galeano H, Sanchis-Gomar F, Fiuza-Luces C, Cristi-Montero C, Bernal-Pino A, Lucia A, Garatachea N (2016). The genetics of exceptional longevity: insights from centenarians. Maturitas..

[13] Dato S, Rose G, Crocco P, Monti D, Garagnani P, Franceschi C, Passarino G (2017). The genetics of human longevity: an intricacy of genes, environment, culture and microbiome. Mech Ageing Dev.

[14] Belloy ME, Napolioni V, Han SS, Le Guen Y, Greicius MD (2020). Alzheimer’s Disease Neuroimaging Initiative. Association of Klotho-VS Heterozygosity With Risk of Alzheimer Disease in Individuals Who Carry APOE4. JAMA Neurol.

[15] Sun L, Hu C, Zheng C, Qian Y, Liang Q, Lv Z, Huang Z, Qi K, Gong H, Zhang Z, Huang J, Zhou Q, Yang Z (2015). FOXO3 variants are beneficial for longevity in southern Chinese living in the Red River Basin: a case-control study and meta-analysis. Sci Rep.

[16] Sun L, Hu CY, Shi XH, Zheng CG, Huang ZZ, Lv ZP, Huang J, Wan G, Qi KY, Liang SY, Zhou L, Yang Z (2013). Trans-ethnical shift of the risk genotype in the CETP I405V with longevity: a Chinese case-control study and meta-analysis. PLoS One.

[17] Huang J, Sun L, Liu M, Zhou L, Lv ZP, Hu CY, Huang ZZ, Zheng CG, Zhou L, Yang Z (2013). Association between SIRT1 gene polymorphisms and longevity of populations from Yongfu region of Guangxi. Zhonghua Yi Xue Yi Chuan Xue Za Zhi.

[18] Fortney K, Dobriban E, Garagnani P, Pirazzini C, Monti D, Mari D, Atzmon G, Barzilai N, Franceschi C, Owen AB, Kim SK (2015). Genome-wide scan informed by age-related disease identifies loci for exceptional human longevity. PLoS Genet.

[19] Sanese P, Forte G, Disciglio V, Grossi V, Simone C (2019). FOXO3 on the road to longevity: lessons from SNPs and chromatin hubs. Comput Struct Biotechnol J.

[20] Willcox BJ, Morris BJ, Tranah GJ, Chen R, Masaki KH, He Q, Willcox DC, Allsopp RC, Moisyadi S, Gerschenson M, Davy PMC, Poon LW, Rodriguez B, Newman AB, Harris TB, Cummings SR, Liu Y, Parimi N, Evans DS, Donlon TA (2017). Longevity-associated FOXO3 genotype and its impact on coronary artery disease mortality in Japanese, whites, and blacks: a prospective study of three American populations. J Gerontol A Biol Sci Med Sci.

[21] Zeng CY, Yang TT, Zhou HJ, Zhao Y, Kuang X, Duan W, Du JR (2019). Lentiviral vector-mediated overexpression of klotho in the brain improves Alzheimer's disease-like pathology and cognitive deficits in mice. Neurobiol Aging.

[22] Zahn JM, Sonu R, Vogel H, Crane E, Mazan-Mamczarz K, Rabkin R, Davis RW, Becker KG, Owen AB, Kim SK (2006). Transcriptional profiling of aging in human muscle reveals a common aging signature. PLoS Genet.

[23] Passtoors WM, Boer JM, Goeman JJ, Akker EB, Deelen J, Zwaan BJ, Scarborough A, Rv B, Vossen RH, Houwing-Duistermaat JJ, Ommen GJ, Westendorp RG, van Heemst D, de Craen AJ, White AJ, Gunn DA, Beekman M, Slagboom PE (2012). Transcriptional profiling of human familial longevity indicates a role for ASF1A and IL7R. PLoS One.

[24] Fushan AA, Turanov AA, Lee SG, Kim EB, Lobanov AV, Yim SH, Buffenstein R, Lee SR, Chang KT, Rhee H, Kim JS, Yang KS, Gladyshev VN (2015). Gene expression defines natural changes in mammalian lifespan. Aging Cell.

[25] Honda Y, Tanaka M, Honda S (2010). Redox regulation, gene expression and longevity. Geriatr Gerontol Int.

[26] Freude S, Schilbach K, Schubert M (2009). The role of IGF-1 receptor and insulin receptor signaling for the pathogenesis of Alzheimer's disease: from model organisms to human disease. Curr Alzheimer Res.

[27] Khan AH, Zou Z, Xiang Y, Chen S, Tian XL (2019). Conserved signaling pathways genetically associated with longevity across the species. Biochim Biophys Acta Mol basis Dis.

[28] Erlandsson MC, Lyngfelt L, Åberg ND, Wasén C, Espino RA, Silfverswärd ST, Nadali M, Jood K, Andersson KME, Pullerits R, Bokarewa MI (2019). Low serum IGF1 is associated with hypertension and predicts early cardiovascular events in women with rheumatoid arthritis. BMC Med.

[29] Maximus PS (2019). Insulin like growth factor 1 is linked to higher cardiovascular risk score in adults with type 2 diabetes mellitus and chronic kidney disease. Diabetes Metab Syndr.

[30] Schneider-Poetsch T, Yoshida M (2018). Along the central dogma-controlling gene expression with small molecules. Annu Rev Biochem.

[31] Chen X, Wang CC, Guan NN (2020). Computational models in non-coding RNA and human disease. Int J Mol Sci.

[32] Xu P, Wang L, Chen D, Feng M, Lu Y, Chen R, Qiu C, Li J (2020). The application of proteomics in the diagnosis and treatment of bronchial asthma. Ann Transl Med.

[33] Yung PYK, Elsässer SJ (2017). Evolution of epigenetic chromatin states. Curr Opin Chem Biol.

[34] Hashimoto H, Vertino PM, Cheng X (2010). Molecular coupling of DNA methylation and histone methylation. Epigenomics..

[35] Morris BJ, Willcox BJ, Donlon TA (2019). Genetic and epigenetic regulation of human aging and longevity. Biochim Biophys Acta Mol basis Dis.

[36] Molina-Serrano D, Kyriakou D, Kirmizis A (2019). Histone modifications as an intersection between diet and longevity. Front Genet.

[37] Huang B, Zhong D, Zhu J, An Y, Gao M, Zhu S, Dang W, Wang X, Yang B, Xie Z (2020). Inhibition of histone acetyltransferase GCN5 extends lifespan in both yeast and human cell lines. Aging Cell.

[38] Rosa-Garrido M, Chapski DJ, Vondriska TM (2018). Epigenomes in cardiovascular disease. Circ Res.

[39] Ibrahim DM, Mundlos S (2020). The role of 3D chromatin domains in gene regulation: a multi-facetted view on genome organization. Curr Opin Genet Dev.

[40] Evans SA, Horrell J, Neretti N (2019). The three-dimensional organization of the genome in cellular senescence and age-associated diseases. Semin Cell Dev Biol.

[41] Bhadra M, Howell P, Dutta S, Heintz C, Mair WB (2020). Alternative splicing in aging and longevity. Hum Genet.

[42] Wei L, Sun J, Zhang N, Zheng Y, Wang X, Lv L, Liu J, Xu Y, Shen Y, Yang M (2020). Noncoding RNAs in gastric cancer: implications for drug resistance. Mol Cancer.

[43] Kinser HE, Pincus Z (2020). MicroRNAs as modulators of longevity and the aging process. Hum Genet.

[44] Shi Z, Zhang K, Zhou H, Jiang L, Xie B, Wang R, Xia W, Yin Y, Gao Z, Cui D, Zhang R, Xu S (2020). Increased miR-34c mediates synaptic deficits by targeting synaptotagmin 1 through ROS-JNK-p53 pathway in Alzheimer’s disease. Aging Cell.

[45] Basisty N, Meyer JG, Schilling B (2018). Protein turnover in aging and longevity. Proteomics..

[46] Hofmann JW, Zhao X, De Cecco M, Peterson AL, Pagliaroli L, Manivannan J, Hubbard GB, Ikeno Y, Zhang Y, Feng B, Li X, Serre T, Qi W, Van Remmen H, Miller RA, Bath KG, de Cabo R, Xu H, Neretti N, Sedivy JM (2015). Reduced expression of MYC increases longevity and enhances healthspan. Cell..

[47] Su L, Chen S, Zheng C, Wei H, Song X (2019). Meta-analysis of gene expression and identification of biological regulatory mechanisms in Alzheimer’s disease. Front Neurosci.

[48] Balashanmugam MV, Shivanandappa TB, Nagarethinam S, Vastrad B, Vastrad C (2019). Analysis of differentially expressed genes in coronary artery disease by integrated microarray analysis. Biomolecules..

[49] Wang W, Liu Q, Wang Y, Piao H, Li B, Zhu Z, Li D, Wang T, Xu R, Liu K (2019). Integration of gene expression profile data of human Epicardial adipose tissue from coronary artery disease to verification of hub genes and pathways. Biomed Res Int.

[50] Wilhelm K, Happel K, Eelen G, Schoors S, Oellerich MF, Lim R, Zimmermann B, Aspalter IM, Franco CA, Boettger T, Braun T, Fruttiger M, Rajewsky K, Keller C, Brüning JC, Gerhardt H, Carmeliet P, Potente M (2016). FOXO1 couples metabolic activity and growth state in the vascular endothelium. Nature..

[51] Barko PC, McMichael MA, Swanson KS, Williams DA (2018). The gastrointestinal microbiome: a review. J Vet Intern Med.

[52] Haran JP, Bhattarai SK, Foley SE, Dutta P, Ward DV, Bucci V, BA MC (2019). mBio.

[53] Kurilshikov A, van den Munckhof ICL, Chen L, Bonder MJ, Schraa K, Rutten JHW, Riksen NP, de Graaf J, Oosting M, Sanna S, Joosten LAB, van der Graaf M, Brand T, Koonen DPY, van Faassen M, Study LLDEEPC, Consortium BBMRIM, Slagboom PE, Xavier RJ, Kuipers F, Hofker MH, Wijmenga C, Netea MG, Zhernakova A, Fu J (2019). Gut Microbial Associations to Plasma Metabolites Linked to Cardiovascular Phenotypes and Risk. Circ Res.

[54] Huang Y, Rosenberg M, Hou L, Hu M (2017). Relationships among environment, climate, and longevity in China. Int J Environ Res Public Health.

[55] Fontana L, Partridge L (2015). Promoting health and longevity through diet: from model organisms to humans. Cell..

[56] Yang Y, Zhang L (2020). The effects of caloric restriction and its mimetics in Alzheimer's disease through autophagy pathways. Food Funct.

[57] Pallazola VA, Davis DM, Whelton SP, Cardoso R, Latina JM, Michos ED, Sarkar S, Blumenthal RS, Arnett DK, Stone NJ, Welty FK (2019). A Clinician’s guide to healthy eating for cardiovascular disease prevention. Mayo Clin Proc Innov Qual Outcomes.

[58] Vatner SF, Zhang J, Oydanich M, Berkman T, Naftalovich R, Vatner DE (2020). Healthful aging mediated by inhibition of oxidative stress. Ageing Res Rev.

[59] Zhang H, Davies KJA, Forman HJ (2015). Oxidative stress response and Nrf2 signaling in aging. Free Radic Biol Med.

[60] Reed M, Best J, Golubitsky M, Stewart I, Nijhout HF (2017). Analysis of homeostatic mechanisms in biochemical networks. Bull Math Biol.

[61] Krycer JR, Quek LE, Francis D, Zadoorian A, Weiss FC, Cooke KC, Nelson ME, Diaz-Vegas A, Humphrey SJ, Scalzo R, Hirayama A, Ikeda S, Shoji F, Suzuki K, Huynh K, Giles C, Varney B, Nagarajan SR, Hoy AJ, Soga T, Meikle PJ, Cooney GJ, Fazakerley DJ, James DE (2020). Insulin signaling requires glucose to promote lipid anabolism in adipocytes. J Biol Chem.

[62] Han S, Schroeder EA, Silva-García CG, Hebestreit K, Mair WB, Brunet A (2017). Mono-unsaturated fatty acids link H3K4me3 modifiers to C. elegans lifespan. Nature..

[63] Wei S, Gao L, Jiang Y, Shang S, Chen C, Dang L, Wang J, Huo K, Wang J, Qu Q (2020). The Apolipoprotein E ε4 allele-dependent relationship between serum lipid levels and cognitive function: a population-based cross-sectional study. Front Aging Neurosci.

[64] Wegner CJ, Kim B, Lee J (2013). Trust your gut: galvanizing nutritional interest in intestinal cholesterol metabolism for protection against cardiovascular diseases. Nutrients..

[65] Campagna J, Spilman P, Jagodzinska B, Bai D, Hatami A, Zhu C, Bilousova T, Jun M, Elias CJ, Pham J, Cole G, LaDu MJ, Jung ME, Bredesen DE, John V (2018). A small molecule ApoE4-targeted therapeutic candidate that normalizes sirtuin 1 levels and improves cognition in an Alzheimer's disease mouse model. Sci Rep.

[66] Hubbard BP, Sinclair DA (2014). Small molecule SIRT1 activators for the treatment of aging and age-related diseases. Trends Pharmacol Sci.

[67] Sebastiani P, Gurinovich A, Nygaard M, Sasaki T, Sweigart B, Bae H, Andersen SL, Villa F, Atzmon G, Christensen K, Arai Y, Barzilai N, Puca A, Christiansen L, Hirose N, Perls TT (2019). APOE alleles and extreme human longevity. J Gerontol A Biol Sci Med Sci.

[68] Sun L, Hu C, Zheng C, Huang Z, Lv Z, Huang J, Liang S, Shi X, Zhu X, Yuan H, Yang Z (2014). Gene-gene interaction between CETP and APOE polymorphisms confers higher risk for hypertriglyceridemia in oldest-old Chinese women. Exp Gerontol.

[69] Lai RW, Lu R, Danthi PS, Bravo JI, Goumba A, Sampathkumar NK, Benayoun BA (2019). Multi-level remodeling of transcriptional landscapes in aging and longevity. BMB Rep.

[70] López-Otín C, Blasco MA, Partridge L, Serrano M, Kroemer G (2013). The hallmarks of aging. Cell..

